# Activated Persulfate Oxidation of Perfluorooctanoic Acid (PFOA) in Groundwater under Acidic Conditions

**DOI:** 10.3390/ijerph13060602

**Published:** 2016-06-17

**Authors:** Penghua Yin, Zhihao Hu, Xin Song, Jianguo Liu, Na Lin

**Affiliations:** 1 College of Engineering and Applied Sciences, Nanjing University, Nanjing 210093, China; phyin@issas.ac.cn (P.Y.); melopasserby@126.com (Z.H.); jgliu2005@163.com (J.L.); 2 Key Laboratory of Soil Environment and Pollution Remediation, Institute of Soil Science, Chinese Academy of Sciences, Nanjing 210008, China; nlin@issas.ac.cn

**Keywords:** PFOA, activated persulfate, free radicals, defluorination

## Abstract

Perfluorooctanoic acid (PFOA) is an emerging contaminant of concern due to its toxicity for human health and ecosystems. However, successful degradation of PFOA in aqueous solutions with a cost-effective method remains a challenge, especially for groundwater. In this study, the degradation of PFOA using activated persulfate under mild conditions was investigated. The impact of different factors on persulfate activity, including pH, temperature (25 °C–50 °C), persulfate dosage and reaction time, was evaluated under different experimental conditions. Contrary to the traditional alkaline-activated persulfate oxidation, it was found that PFOA can be effectively degraded using activated persulfate under acidic conditions, with the degradation kinetics following the pseudo-first-order decay model. Higher temperature, higher persulfate dosage and increased reaction time generally result in higher PFOA degradation efficiency. Experimental results show that a PFOA degradation efficiency of 89.9% can be achieved by activated persulfate at pH of 2.0, with the reaction temperature of 50 °C, molar ratio of PFOA to persulfate as 1:100, and a reaction time of 100 h. The corresponding defluorination ratio under these conditions was 23.9%, indicating that not all PFOA decomposed via fluorine removal. The electron paramagnetic resonance spectrometer analysis results indicate that both SO_4_^−^• and •OH contribute to the decomposition of PFOA. It is proposed that PFOA degradation occurs via a decarboxylation reaction triggered by SO_4_^−^•, followed by a HF elimination process aided by •OH, which produces one-CF_2_-unit-shortened perfluoroalkyl carboxylic acids (PFCAs, C_n−1_F_2n−1_COOH). The decarboxylation and HF elimination processes would repeat and eventually lead to the complete mineralization all PFCAs.

## 1. Introduction

Perfluorooctanoic acid (PFOA) is one of the most important perfluorochemicals (PFCs) due to its extensive applications in electronics, chemical, medical and manufacturing industries (e.g., as a surfactant, surface treatment agent, polymer, metal coating, and fire retardant), which result in its release from a wide range of commercial products [1,2]. Because of its toxicity, potential for bioaccumulation, and common occurrence in water resources, PFOA has been recognized as an emerging environmental pollutant [3]. PFOA occurrence has been observed worldwide. For example, Arias *et al.* observed PFOA concentrations ranging from 0.6 to 1.7 milligrams per liter (mg/L) in an evaporation pond used to collect wastewater generated from fire-fighting exercises at a military air base [1]. In the surface water of a Canadian tributary, PFOA was reported at concentrations up to 11.3 micrograms per liter (μg/L) due to a release of fire-fighting foams [4]. PFOA was also detected in groundwater at Washington county landfill at 41.6 μg/L [5]. These contamination in wastewater, surface water and groundwater leads to the PFOA detection in drinking water. For example, in West Virginia and Ohio, PFOA was detected at levels of up to 13 μg/L in private water supply wells [6]. The United States Environmental Protection Agency (USEPA) issued the provisional health advisory (PHA) value for drinking water of 400 nanograms per liter (ng/L) for PFOA in January 2009 [7]. An advisory threshold value of 300 ng/L for the sum of PFOA and PFOS (perfluorooctane sulfonate) has also been set by the German drinking water commission [8]. Comparison of observed PFOA levels in the environment to drinking water advisory levels indicates an urgent need to develop effective methods to degrade PFOA in groundwater so as to protect human health and ecosystems. 

However, PFOA is chemically inert and recalcitrant to most chemical and microbiological treatment [9,10,11,12] due to its high-energy carbon-fluorine bonds. A number of photolytic methods, such as direct photolysis [13,14,15], persulfate photolysis [14,16,17,18], alkaline isopropanol photolysis [19] and photocatalysis [20,21,22], have shown varying degrees of efficacy in the degradation of PFOA. However, these methods are generally very costly and difficult to implement in the field. Advanced oxidation processes (AOPs), which utilize strong oxidants to produce free radicals, have been used widely for in-situ chemical oxidation (ISCO) of a wide range of organic pollutants, showing potential ability to degrade PFOA in groundwater. Fenton’s reagent (*i.e.*, H_2_O_2_ and Fe^2+^ salts), alkaline ozonation and peroxone (*i.e.*, a mixture of O_3_ and H_2_O_2_), utilizing the hydroxyl radical in AOPs, have been shown to be relatively ineffective for PFOA destruction [13,23,24]. 

Persulfate (S_2_O_8_^2−^), a strong and relatively stable oxidant (E_0_ = 2.01 eV), can be activated to generate free radicals to achieve a higher oxidative potential under conditions of heat [25], light [26], or chemical activation (e.g., by a base or metal ions) [27,28,29]. The persulfate anion can produce SO_4_^−^• (E_0_ = 2.6 V), •OH (E_0_ = 2.7 V) and O_2_^−^• (E_0_ = −0.33 V) [30,31,32], resulting in its highly reactive radical species for degrading different organic contaminants after activation. Early studies have shown that persulfate can be used to degrade PFOA with different activation methods such as photolysis [17,18,19], UV [14,16,17,18] and microwave [33]. Heat-activated (85 °C) persulfate oxidation has been reported to transform PFOA in water to fluoride and CO_2_ [34]. However, these extreme activation conditions limit the practical application in PFOA remediation, especially for the remediation of PFOA in groundwater. In addition, these processes are energy-intensive, hence very expensive to implement in full-scale applications. 

Little research has been reported on the degradation of PFOA by persulfate under mild conditions. Celyna *et al.* reported a low degradation efficiency of PFOA by persulfate using iron-modified diatomite under alkaline conditions [35]. Lee *et al.* observed some PFOA degradation by persulfate under acidic conditions [36], showing a potential approach to remediate PFOA contamination in groundwater, but the effective radicals responsible for effective degradation of PFOA were not identified and the degradation mechanisms of degradation were not clear. Therefore, optimal conditions for PFOA degradation by persulfate under mild conditions, along with the PFOA degradation mechanisms, need to be further investigated. The primary goal of this study was to investigate the oxidative degradation of PFOA in groundwater by activated persulfate under mild conditions, which is feasible to implement in full-scale applications. Experiments were conducted to: (i) evaluate the influence of pH, temperature, persulfate dosage and reaction time on the PFOA degradation efficiency under mild conditions; (ii) identify the radicals that are effective in degrading PFOA; and (iii) determine the degradation kinetics and explore the degradation pathway of PFOA via intermediate analyses.

## 2. Materials and Methods

### 2.1. Chemical Reagents

PFOA (C_7_F_15_COOH, molecular weight 414.07, 96% purity) and the free radical spin trapping reagent 5,5-dimethyl-1-pyrrolidine N-oxide (DMPO, 97%) were purchased from Aladdin (Georgetown, TX, USA). The rest of the chemical reagents used in this study, including sodium persulfate (Na_2_S_2_O_8_, 99%), sulfuric acid (H_2_SO_4_, 97%), hydrochloric acid (HCl, 36%), sodium hydroxide (NaOH, 96%), sodium fluoride (NaF, 98%), potassium phosphate dibasic anhydrous (K_2_HPO_4_, 98%), potassium dihydrogen phosphate (KH_2_PO_4_, 99.5%), trisodium citrate dihydrate (C_6_H_5_Na_3_O_7_·2H_2_O, 99%), methanol (CH_4_O, 99.7%) and ethanol (C_2_H_6_O, 99.7%) were of analytical grade and purchased from Sinopharm Chemical Reagent Corporation Ltd. (Beijing, China). The deionized water used in all experiments, with a resistivity of 18.0 MΩ cm, was purified by a Milli-Q system. 

### 2.2. Experimental Methods

Batch experiments to evaluate the influence of different experimental factors (including pH, persulfate dosage, temperature and reaction times) on PFOA degradation efficiency were carried out in 40-mL borate glass bottles. Due to the significance of pH in the persulfate activation under mild conditions, experiments were conducted first to study the effect of pH. In these experiments, pH of solutions was adjusted by adding NaOH or H_2_SO_4_ unless specified otherwise (e.g., HCl). In subsequent experiments, pH of solutions was adjusted to 2.0 by H_2_SO_4_, the initial concentration of PFOA was 20 μM, and initial persulfate concentrations ranged from 0.5 mM to 4 mM. The containers were then placed into constant-temperature water baths without shaking (T = 25, 35, 40 and 50 °C). At the designated sampling times, an appropriate amount of ethanol (molar ratio of ethanol and persulfate was 100:1) was added to quench the reaction immediately. Samples were then taken and preserved at 4 °C in the refrigerator before analysis. All experiments were conducted in triplicate.

Experiments for PFOA kinetics study were conducted under acidic conditions, with pH adjusted by H_2_SO_4_ to 2.0. In these experiments, PFOA (20 μM) and persulfate (2 mM) were mixed to react at different temperature: 30 °C, 40 °C and 50 °C. At the designated sampling times, an appropriate amount of ethanol was added and samples were taken and preserved at 4 °C in the refrigerator. For PFOA degradation byproducts analyses, PFOA (20 μM) and persulfate (2 mM) were mixed to react at a temperature of 50 °C, with pH adjusted by H_2_SO_4_ to 2.0. After a reaction time of 100 h, ethanol was added to quench the reaction immediately and samples were taken for analysis.

Free radical analyses, which were used to identify the effective radicals contributing to the degradation of PFOA, were conducted under three different experimental conditions: (1) PFOA only in aqueous solution as the control system; (2) PFOA with persulfate under acidic conditions; and (3) PFOA with persulfate under alkaline conditions. In the control system, 25 mL of PFOA at a concentration of 20 μM was added to the 40-mL borate glass bottles at a temperature of 50 °C. The solution pH was not adjusted and the final pH was 4.2. For experiments under acidic conditions and alkaline conditions, 20 μM PFOA and 2 mM persulfate were mixed to react at a temperature of 50 °C, with their solution pH adjusted by H_2_SO_4_ to 2.0 and NaOH to 12, respectively. After a reaction time of 10 min, an appropriate amount of DMPO (*i.e.*, that would result in a final concentration of 100 mM DMPO in the system), dissolved in phosphate buffer at pH 7.4, was added immediately to react with free radicals produced by persulfate. 20 μL of the mixed solution were then loaded in the quartz capillary tube of EPR for analysis.

### 2.3. Analytical Methods

Concentrations of PFOA and its degradation byproducts were analyzed by a high-performance liquid chromatograph (HPLC), using an ACQUITY UPLC system (Waters, Milford, MA, USA) equipped with a Waters ACQUITY TQD triple quadrupole mass spectrometer. All samples were extracted using solid phase extraction (SPE) without prefiltration. The Oasis WAX cartridges (6 cubic centimeter, 150 mg, 30 μm) were cleaned and conditioned with 4 mL of 0.1% NH_4_OH in methanol, 4 mL of methanol, and 4 mL of Milli-Q water. The cartridges were washed with 4 mL of buffer solution (25 mM acetic acid/ammonium acetate, pH = 4), centrifuged for 3 min at 3000 revolutions/min to remove residual water, and then washed with 4 mL of methanol. Target compounds were then eluted with 4 mL of 0.1% NH_4_OH in methanol. The latter fraction was concentrated to 0.5 mL using a bath-typed nitrogen blowing instrument and then passed through a polypropylene-membrane syringe filter (Acrodisc GHP, 13 mm, 0.2 μm). 100 μL of filtrate and 100 μL of Milli-Q water were transferred into a polypropylene vial for concentration analysis. Further details of the analysis method, such as column installation, column temperature, mobile phase and its flow rate, can be found in the study by Yu *et al.* [37]. PFOA and its degradation byproducts recoveries under the different analytical conditions were 77%–105%, and the quantitative detection limit for PFOA was 0.10 ng/L. 

Concentrations of fluoride ion were analyzed using a Dionex-500 ion chromatography system (Dionex, Sunnyvale, CA, USA). The samples were filtered through Dionex OnGuard cartridges (1 cubic centimeter; Dionex) to reduce the interference of organic compounds before analysis. The cartridges were cleaned and conditioned with 4 mL of methanol and 10 mL of Milli-Q water before use. Fluoride analysis was carried out using an AS14A (4 mm × 250 mm) analytical column and Ionpac AG14A 4-mm guard column (4 mm × 50 mm) with a degassed 8.0 mM sodium carbonate and 1.0 mM sodium bicarbonate mobile phase at a flow rate of 1 mL·min^−1^. The retention time for fluoride ion was 3.12 min. In addition, a fluoride-ion-selective electrode was also used to analyze concentrations of fluoride ion, after a 1:1 dilution with an ionic strength adjustment buffer. The ionic strength adjustment buffer solutions were prepared by adding hydrochloric acid into trisodium citrate dehydrate aqueous solution (1 M) adjusting solution pH to 6.00. The quantitative detection limits were 1 μg/L and 20 μg/L, for the ion chromatography and ion selective electrode, respectively.

An electron paramagnetic resonance (EPR) spectrometer was used to analyze free radicals generated by activated persulfate. The EPR spectra were obtained using a Bruker-EMX-10/12 spectrometer (Bruker, Karlsruhe, Germany) with a magnetic field modulation frequency of 9.77 GHz, microwave power of 19.97 mW, modulation frequency of 100 kHz, modulation amplitude of 2.0 G, sweep width of 100 G, time constant of 40.95 ms, sweep time of 83.85 s, and receiver gain of 2.0 × 10^4^.

## 3. Results and Discussion

The defluorination ratio (DF ratio), expressed as (moles of fluorine released due to PFOA degradation)/(total moles of fluorine in initial PFOA solution), is an indicator of PFOA degradation and was used to evaluate the defluorination efficiency of PFOA by activated persulfate. PFOA degradation efficiency, expressed as (moles of PFOA degraded)/(moles of initial PFOA concentration), was also used in the evaluation of PFOA removal efficiency by activated persulfate.

### 3.1. Effect of pH

**Figure 1:**
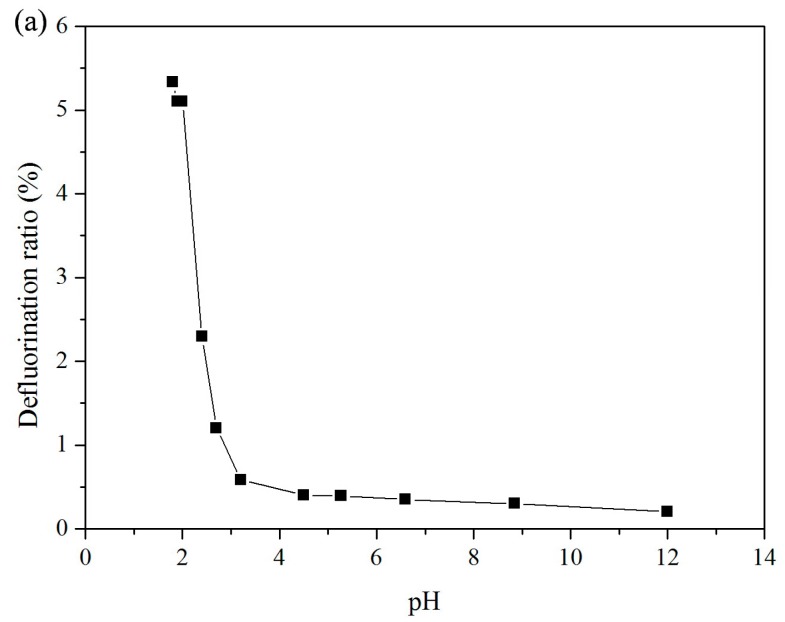

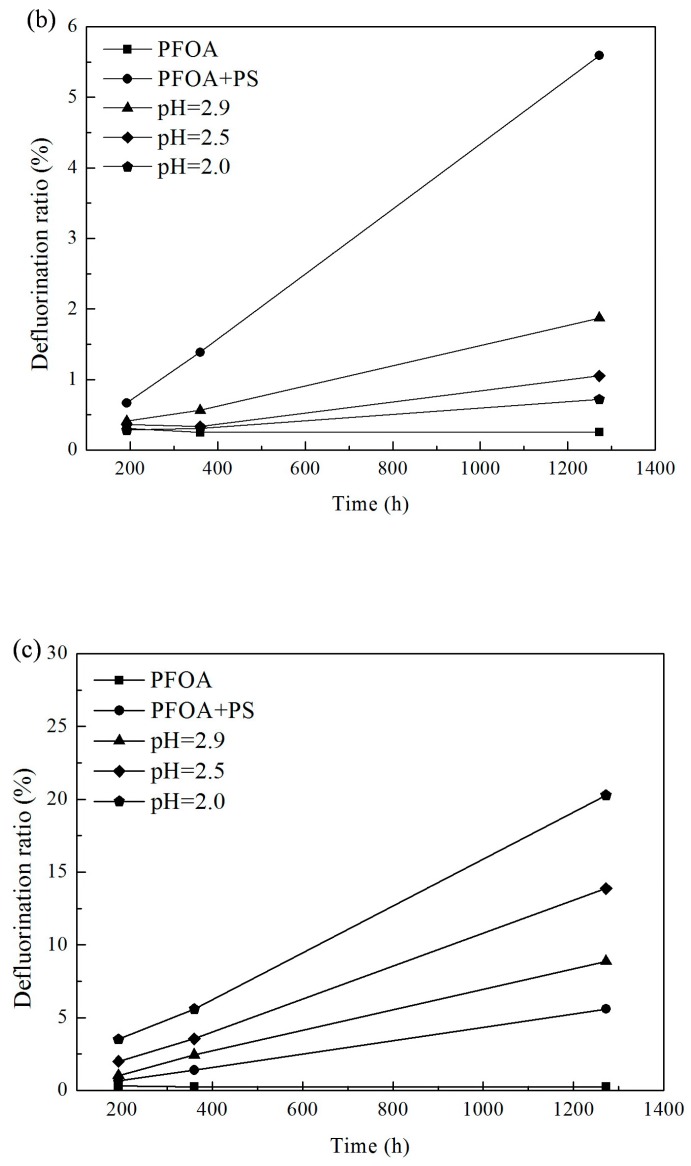
Effects of pH on the degradation of PFOA: (**a**) Solution pH was adjusted by NaOH and H_2_SO_4_ from 1.8 to 12.0, [PFOA]_0_ = 20 μM; [persulfate]_0_ = 2 mM, reaction temperature: 40 °C, reaction time: 22 h; (**b**) solution pH adjusted by HCl, room temperature (<20 °C), [PFOA]_0_ = 20 μM, [persulfate]_0_ = 2 mM; and (**c**) solution pH adjusted by H_2_SO_4_, reaction temperature (at room temperature < 20 °C), [PFOA]_0_ = 20 μM, [persulfate]_0_ = 2 mM. The initial pH value of 20 μM PFOA was about 5.4, and the initial pH value of 20 μM PFOA with 2 mM persulfate, without pH adjustment, was about 4.2.

The effects of pH on PFOA degradation, with a molar ratio of 1:100 PFOA to persulfate, are illustrated in Figure 1. The pH of the solutions was adjusted by adding H_2_SO_4_ and NaOH (Figure 1a), HCl (Figure 1b) and H_2_SO_4_ (Figure 1c), respectively. The initial pH value of 20 μM PFOA and 2 mM persulfate, without pH adjustment, was about 4.2. The experimental results show that solution pH plays a major role in the defluorination efficiency of PFOA. In solutions where pH was adjusted by H_2_SO_4_ and NaOH, the decrease in solution pH (from 12.0 to 2.0) resulted in an increase in the DF ratio (Figure 1a). DF ratios were significantly higher in solutions with pH values less than 3. According to an early study by Liang and Lee [38], in activated persulfate solutions with varying pH, SO_4_^−^• is the predominant oxidant radical at acid pH (e.g., pH < 3); both SO_4_^−^• and •OH are present at neutral pH; and at pH > 12, •OH is the predominant free radical, and reductant radical O_2_^−^• may also be present. The higher DF ratio under acidic conditions (pH < 3) could be due to the production of predominant SO_4_^−^•, as explained through a proton-catalyzing process to form sulfate free radicals as shown in Equations (1) and (2) [36]. The measured pH value in solution decreased from 4.2 to 2.4 after a reaction time of 100 h, consistent with the proton production in Equation (2). At pH > 5, however, OH^−^ may act as a fast scavenger, converting SO_4_^−^• to •OH through the path demonstrated in Equation (3), considering the reaction rate of Equation (3) is much higher than that of Equation (4) [36]. The much lower DF ratio observed at pH > 5 suggests that the SO_4_^−^• free radical plays a major role in PFOA degradation, and •OH itself cannot oxidize PFOA directly [13,39].

(1) $$S_{2}O_{8}^{2-}+H^{+}\to {\text{HS}}_{2}O_{8}^{-}$$

(2) $${\text{HS}}_{2}O_{8}^{-}\to {\text{SO}}_{4}^{2-}+{\text{SO}}_{4}^{-}•+H^{+}$$

(3) $${\text{Alkaline pH}:\text{SO}}_{4}^{-}•+{\text{OH}}^{-}\to {\text{SO}}_{4}^{2-}+•\text{OH},\;k=7\times 10^{-7}\;M^{-1}\cdot s^{-1}$$

(4) $${\text{AlL pHs}:\text{SO}}_{4}^{-}•+H_{2}O\to {\text{SO}}_{4}^{2-}+•{\text{OH}+H}^{+},\;k<60\;M^{-1}\cdot s^{-1}$$

In addition, it was noted that the acid reagent (H_2_SO_4_ *versus* HCl) used to adjust the solution pH also affected the DF ratio. When solution pH was adjusted by HCl, the DF ratio was much lower (data shown in Figure 1b) than when H_2_SO_4_ was used for pH adjustment (data shown in Figure 1c). Therefore, further experimentation, with the total reaction time greater than 1200 h, was carried out to investigate the cause of this phenomenon. As shown in Figure 1b, the DF ratios were less in solutions where pH was adjusted by HCl (pH < 3) than in those where pH was not adjusted (with an initial pH of 4.2). Furthermore, it was noted that, due to the low reactivity in solutions with pH adjusted by HCl, the DF ratio was less than 1% at pH 2.0 after 1200 h (Figure 1b). In contrast, for the solution with pH adjusted by H_2_SO_4_, the DF ratio was more than 20% at pH 2.0. This may be attributed to the presence of chloride ion, which can annihilate reactive free radicals as demonstrated in Equations (5)–(7) [40,41]. This is consistent with results from early studies, which showed that the chloride anion can act as a free radical scavenger, thus inhibiting the reaction between PFOA and free radicals [42].

(5) $${\text{Cl}}^{-}+{\text{SO}}_{4}^{-}•\to {\text{SO}}_{4}^{2-}+\text{Cl}•$$

(6) $${\text{Cl}}^{-}+•\text{OH}\to {\text{ClOH}}^{•-}$$

(7) $${\text{ClOH}}^{•-}+H^{+}\to H_{2}O+\text{Cl}•$$

Based on the results discussed above, pH for subsequent experiments (*i.e.*, to study the effect of persulfate dosage, temperature, and reaction time) was adjusted by H_2_SO_4_ to 2.0 in an effort to obtain optimal experimental conditions for PFOA removal. Effects of persulfate dosage, temperature and reaction time on PFOA degradation by persulfate under acidic conditions were further explored.

### 3.2. Effect of Persulfate Dosage

**Figure 2:**
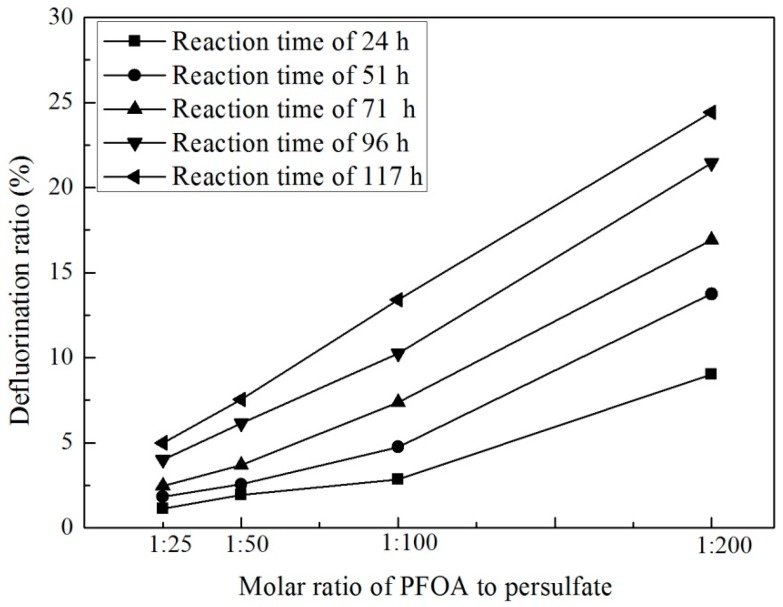
Comparison of PFOA degradation given different concentrations of persulfate ([PFOA]_0_ = 20 μM; temperature = 40 °C; molar ratio of PFOA to persulfate are 1:25, 1:50, 1:100 and 1:200; pH adjusted by H_2_SO_4_ to 2.0).

The impact of persulfate dosage on the DF ratio was studied following those preliminary experiments on the influence of pH. As shown in Figure 2, the DF ratio increased with both increasing persulfate dosage and reaction time at 40 °C. At the higher persulfate dosages (molar ratios of PFOA to persulfate of 1:100 and 1:200), DF ratios could exceed 10% and were as high as approximately 25% in the case of a 1:200 molar ratio (PFOA to persulfate) and after a reaction time of 117 h. This indicates that excessive persulfate is required for effective PFOA degradation. This observation has significant ramifications on the engineered application of activated persulfate to remediate PFOA since insufficient persulfate dosage may result in the failure of PFOA remediation in the field.

### 3.3. Effect of Temperature and Reaction Time

**Figure 3:**
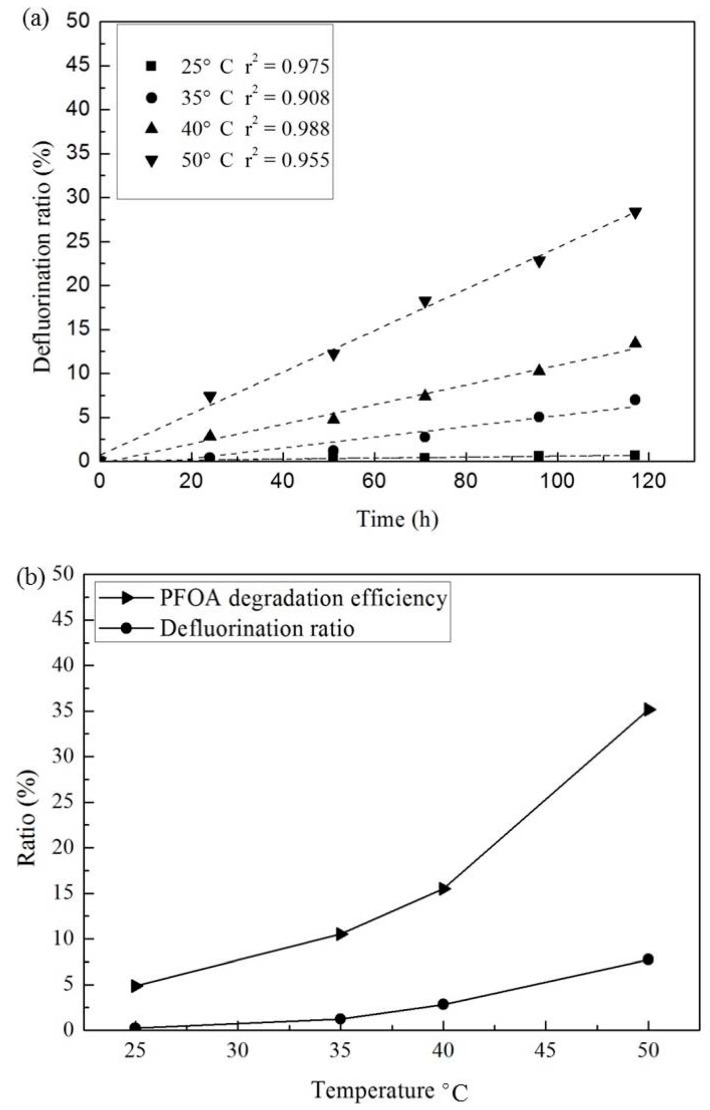

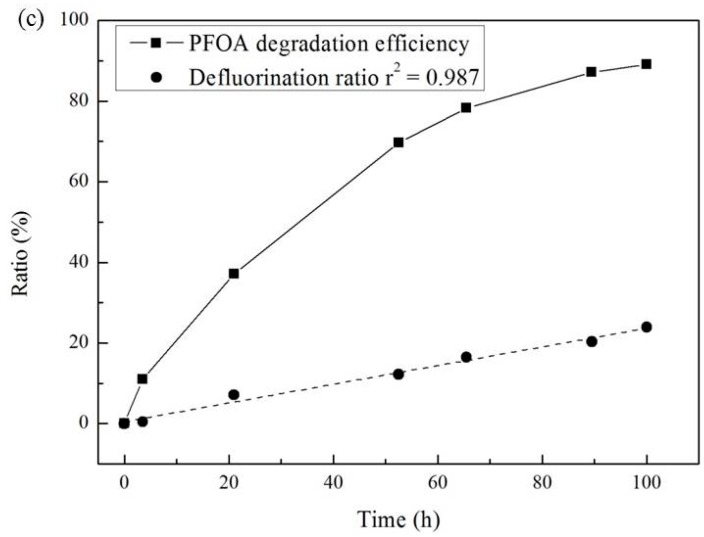
Effect of temperature and reaction time on PFOA degradation: (**a**) effect of reaction time on PFOA DF ratio at different temperature ([PFOA]_0_ = 20 μM; the molar ratio of PFOA to persulfate = 1:100; temperature = 25 °C, 35 °C, 40 °C, 50 °C; pH adjusted by H_2_SO_4_ to 2.0); and (**b**) effect of temperature on PFOA degradation efficiency and DF ratio ([PFOA]_0_ = 20 μM; molar ratio of PFOA to persulfate = 1:100; reaction time = 24 h; pH adjusted by H_2_SO_4_ to 2.0); and (**c**) effect of reaction time on PFOA degradation efficiency and DF ratio ([PFOA]_0_ = 20 μM; molar ratio of PFOA to persulfate = 1:100; temperature = 50 °C; pH adjusted by H_2_SO_4_ to 2.0).

Additional experiments were conducted to evaluate the effect of temperature on PFOA degradation by activated persulfate under acidic conditions. The results are shown in Figure 3. The temperature range tested included 25 °C, 35 °C, 40 °C and 50 °C. As expected, the DF ratio increased with increasing temperature, and the incremental increase in DF ratio was more significant when temperatures were higher than 40 °C (Figure 3a). This is due to the influence of heat activation as shown in Equation (8), and is consistent with the literature which has demonstrated that heat-activation of persulfate can be achieved at temperatures above 40 °C [25]. The impact of temperature was further evaluated using the solution system with a molar ratio of PFOA to persulfate of 1:100 and with a reaction time of 24 h at 50 °C (Figure 3b). The corresponding DF ratio was 7.45%, which accounts for approximately 35.7% of the PFOA degradation efficiency. This suggests that not all PFOA degrades via fluorine removal.

(8) $$S_{2}O_{8}^{2-}+\text{heat}\to 2{\text{SO}}_{4}^{-}•$$

The effect of reaction time was also explored, and the results are shown in Figure 3a,c. As shown in Figure 3a, the DF ratio increased with increasing reaction time. The r^2^ value of the linear regression fitting of DF ratio to reaction time varied from 0.908 to 0.988, demonstrating a linear relationship between DF ratio and reaction time through the reaction period until a DF ratio of 25% was achieved. As with the effect of temperature, this observation was further verified by experiments conducted at a temperature of 50 °C (Figure 3c). Both the PFOA degradation efficiency and DF ratio increased with increasing reaction time. The DF ratio exhibited a linear relationship with reaction time, and the r^2^ value of the linear regression fitting was 0.987. In contrast, the PFOA degradation efficiency initially increased faster than the DF ratio, and then slowed down after a reaction time of 80 h when the degradation efficiency was up to 80%. After a reaction time of 100 h, the DF ratio was 23.9%, accounting for 89.9% of PFOA degraded. This indicates that PFOA degraded by activated persulfate does not undergo complete conversion to fluorine and carbon dioxide directly, but rather undergoes a stepwise degradation process. Even though the long reaction time makes it more difficult to implement a large-scale remediation system for PFOA removal from groundwater, this finding is very helpful for engineers who are in charge of determining the monitoring frequency to evaluate the effectiveness of PFOA remediation with activated persulfate. 

**Figure 4:**
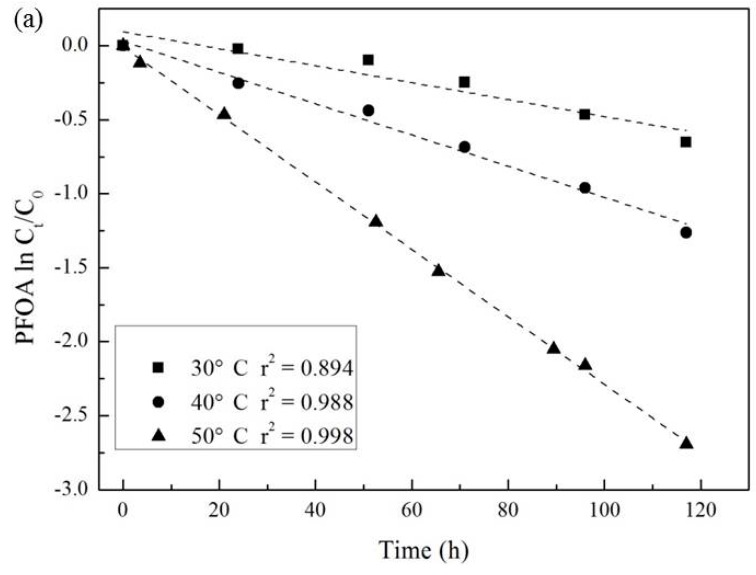

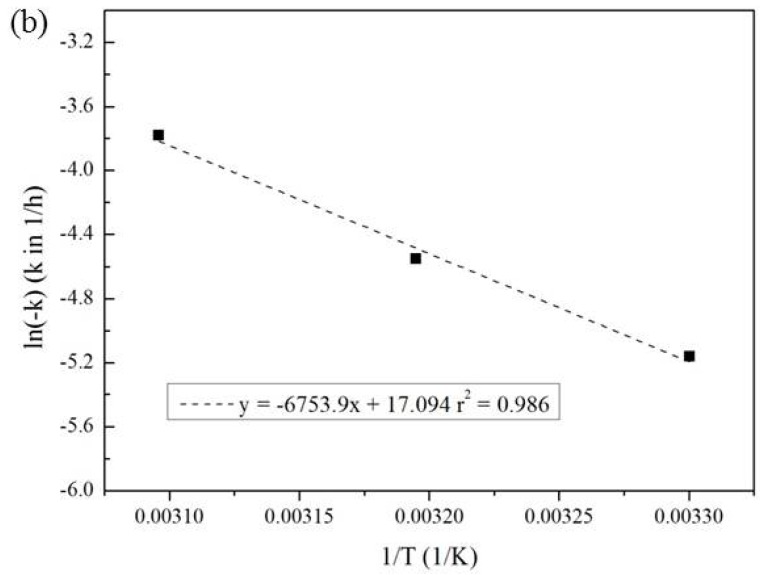
(**a**) The pseudo-first-order kinetic plots of PFOA degradation by activated persulfate under acidic conditions ([PFOA]_0_ = 20 μM; molar ratio of PFOA to persulfate = 1:100; temperature = 30 °C, 40 °C, 50 °C; pH adjusted by H_2_SO_4_ to 2.0); (**b**) Arrhenius plot of the pseudo-first-order degradation constants.

The kinetics of PFOA degradation by activated persulfate under acidic conditions are shown in Figure 4a, where PFOA concentration changes were plotted as ln(C_t_/C_0_) *versus* reaction time at temperature of 30 °C, 40 °C and 50 °C. The degradation of PFOA by activated persulfate under acidic conditions was found to follow the pseudo-first-order decay model, with the *r*^2^ correlation values of 0.894, 0.988 and 0.998, for temperature of 30 °C, 40 °C and 50 °C, respectively. 

Table 1 shows the fitted kinetic constants (k), and the corresponding half-lives (t_1/2_), which can be used to evaluate the degradation rate at different temperature. The k values of PFOA degradation by activated persulfate under acidic conditions increase with increasing temperature, further demonstrating the significant effect of temperature on PFOA degradation. 

Figure 4b showed an Arrhenius plot of the pseudo-first-order degradation constants associated with three different temperature. According to the Arrhenius Equation (9), the activation energy (Ea) for the degradation of PFOA by activated persulfate under acidic conditions was determined to be 56.2 kJ/mol (*r*^2^ = 0.986), which is in good agreement with previous study by Lee *et al.* [36]. This apparent activation energy is lower than those for the oxidation of chlorinated ethenes by heat-activated persulfate (97–143 kJ/mol) [43], indicating that the apparent activation energy only accounts for the one decarboxylation step, which results in PFOA degradation. This suggests that PFOA degradation by activated persulfate undergoes a stepwise degradation process, as discussed in more detail later.

(9) $$\text{lnk}=\text{lnA}-\frac{Ea}{RT}$$

### 3.4. Reaction Mechanisms

**Figure 5:**
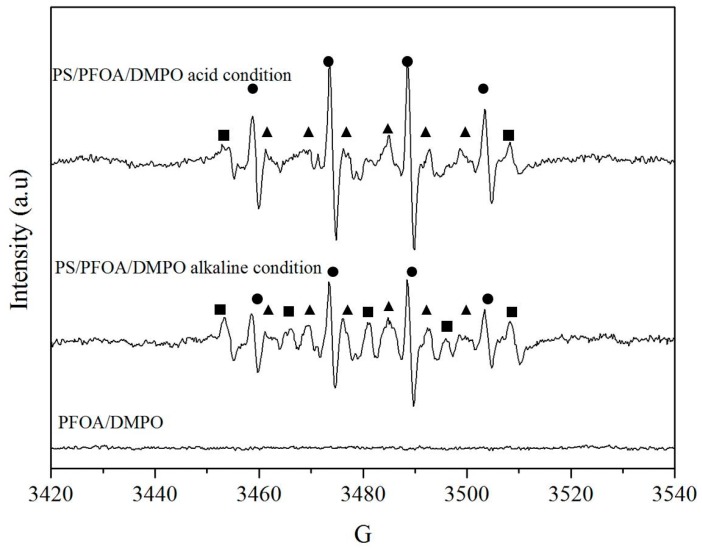
EPR spectra of radical adducts as a function of pH. Experimental conditions: solution pH was adjusted by H_2_SO_4_ and NaOH. [PFOA]_0_ = 20 μM; [persulfate]_0_ = 2 mM; temperature = 50 °C; reaction time = 10 min; [DMPO] = 100 mM. Circle represents the signal of DMPO–OH, triangle represents the signal of DMPO-SO_4_ and square represents signals of unidentified radicals.

In order to further understand PFOA degradation mechanisms under acidic conditions, free radicals analysis experiments were carried out along with quantification of PFOA and its daughter products. Figure 5 shows DMPO hydroxyl radicals adduct (DMPO-OH) with hyperfine splitting constants of aN = 14.9 and aHβ = 14.8, and DMPO sulfate radicals adduct (DMPO-SO_4_) with hyperfine splitting constant of aN = 13.51, aHβ = 9.91, aH_γ1_ = 1.34 and aH_γ2_ = 0.88 in the EPR spectra for different solution systems in which pH was adjusted by H_2_SO_4_ and NaOH. The EPR spectra of radical adducts show the generation of •OH and SO_4_^−^• in solutions under both acidic and alkaline conditions. Relatively little degradation of PFOA was observed in persulfate solution systems with high pH values. According to Equation (3), SO_4_^−^• reacts rapidly with OH^−^ to produce •OH under alkaline conditions. This suggests that •OH itself may not be able to degrade PFOA, consistent with the conclusion of early studies that showed •OH cannot oxidize PFOA directly [23,44]. 

The predominant SO_4_^−^• produced by protons under acidic conditions (pH < 3), as discussed earlier, plays a significant role in the successful degradation of PFOA. It was speculated that the proton increases the reaction rate of sulfate radicals with PFOA, which leads to the efficient degradation of PFOA. Furthermore, consistent with the results of a previous study [45], the signal intensity of DMPO-OH adduct was much stronger than that of DMPO-SO_4_. A possible explanation is the radical conversion as described in Equations (10) and (11) [46].

(10) $${2S}_{2}O_{8}^{2-}+2H_{2}O\to 3{\text{SO}}_{4}^{2-}+{\text{SO}}_{4}^{-}•+O_{2}+4H^{+}$$

(11) $${\text{SO}}_{4}^{-}•+{\text{OH}}^{-}\to {\text{SO}}_{4}^{2-}+•\text{OH}$$

Furthermore, concentrations of PFOA and its daughter products were quantified, after a reaction time of 100 h, and the results are summarized in Table 2. 

It was observed that, in general, the total molarity of perfluoroalkyl carboxylic acid (PFCA) intermediates was equal to the initial concentration of PFOA. As indicated by the earlier conclusion that not all PFOA decomposes via fluorine removal (as seen from the discrepancy between DF ratio and degradation efficiency), it can be deduced that concentrations of shorter-chain PFCs increase with increased reaction time. The analysis results indicate a stepwise degradation process, consistent with those reported in early studies [33,36]. 

**Figure 6:**
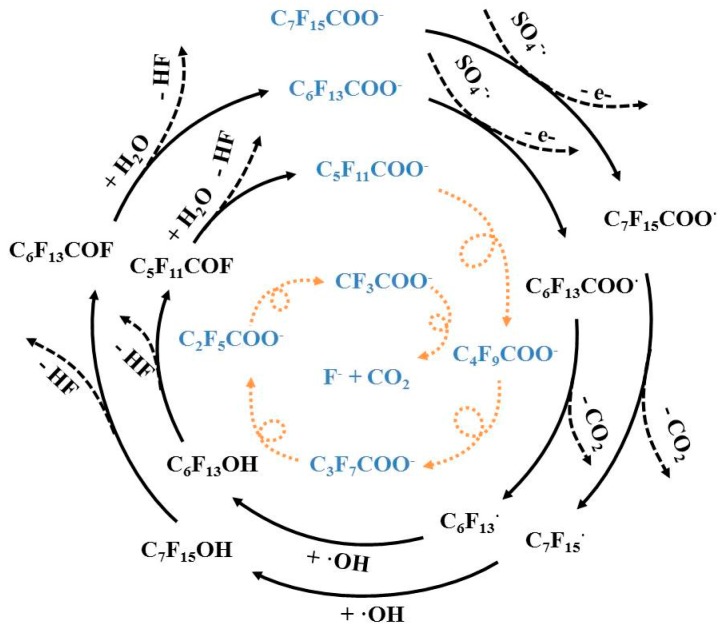
Schematic pathway for PFOA degradation (mainly pathway).

Considering the free radical and degradation byproduct analyses discussed above, together with the mechanisms of PFOA degradation reported previously [47,48], a possible chemical reaction pathway for PFOA oxidation by activated persulfate under acidic conditions can be described by Equation (12) through Equation (16) below and is shown in Figure 6. As a first step, one electron is transferred from PFOA to reactive radical SO_4_^−^• to initiate a decarboxylation reaction and perfluoroalkyl radicals are formed (*i.e.*, C_n_F_2n+1_•). The newly formed C_n_F_2n+1_• continues to react with •OH to form C_n_F_2n+1_OH, which undergoes an HF elimination to form C_n−1_F_2n−1_COF [49], resulting in the formation of one-CF_2_-unit-shortened PFCA (C_n−1_F_2n−1_COOH) *via* hydrolysis [50]. The decarboxylation and HF elimination processes continue until all PFCAs are converted to CO_2_ and fluoride (Figure 6).

(12) $${\text{SO}}_{4}^{-}•+C_{7}F_{15}{\text{COO}}^{-}\to {\text{SO}}_{4}^{2-}+C_{7}F_{15}\text{COO}•$$

(13) $$C_{7}F_{15}\text{COO}•\to {\text{CO}}_{2}+C_{7}F_{15}•$$

(14) $$C_{7}F_{15}•+•\text{OH}\to C_{7}F_{15}\text{OH}$$

(15) $$C_{7}F_{15}\text{OH}\to C_{6}F_{13}{\text{COF}+F}^{-}{+H}^{+}$$

(16) $$C_{6}F_{13}\text{COF}+H_{2}O\to C_{6}F_{13}{\text{COO}}^{-}+F^{-}+2H^{+}$$

## 4. Conclusions

This study demonstrates that advanced chemical oxidation of PFOA by activated persulfate under acidic conditions in aqueous solutions is effective, while its degradation by the traditional alkaline-activated persulfate shows rather low degradation efficiency. Experimental results showed that higher temperature, higher persulfate dosage and a longer reaction time resulted in higher PFOA degradation efficiency by activated persulfate under acidic conditions. The degradation kinetics of PFOA was found to follow the pseudo-first-order decay model. It was demonstrated that 89.9% of PFOA can be degraded after a reaction time of 100 h in a solution system with pH 2.0 at a temperature of 50 °C. The corresponding DF ratio of 23.9% was much less than the PFOA degradation efficiency, indicating that not all PFOA degradation is achieved via fluorine removal. EPR data showed that SO_4_^−^•, along with •OH, are the effective free radicals contributing to PFOA degradation. It was speculated that SO_4_^−^• initiated a decarboxylation reaction in PFOA, followed by HF elimination due to •OH, and formed one-CF_2_-unit-shortened PFCA (C_n−1_F_2n−1_COOH). The discoveries of this study provide a basis for the cost-effective remediation approach of PFOA contamination in aqueous solutions, especially in groundwater. However, it is still technically challenging due to the fact that remediation at elevated temperature is difficult to implement in the field at large scale. Hence, further study to improve the efficiency of sulfate radical production to accelerate the degradation of PFOA and its associated intermediate products is needed. In addition, the influence of soil on the PFOA degradation by activated persulfate under acidic conditions was not investigated in this study. Further study to evaluate the impact of soil will prove to be valuable. 

## Figures and Tables

**Table 1:** Pseudo-first-order kinetic constant (k) of PFOA degradation by activated persulfate under different reaction temperatures, ([PFOA]_0_ = 20 μM; [persulfate]_0_ = 2 mM; pH adjusted by H_2_SO_4_ to 2.0).

Temperature (°C)	Pseudo-First-Order Kinetic Constant k (10^−2^·h^−1^)	t_1/2_ (h)	*r*^2^
30	0.57	138	0.894
40	1.06	69	0.988
50	2.28	30	0.998

**Table 2:** Concentrations of PFCA intermediates formed by PFOA (20 μM) with a reaction time of 100 h, 2 mM persulfate, initial pH = 2.0 adjusted by H_2_SO_4_, temperature = 50 °C.

PFCA Intermediates	Concentrations (μmol/L)
PFOA, C8	1.93
Perfluoroheptanoic acid, PFHpA, C7	5.91
Perfluorohexanoic acid, PFHeA, C6	3.82
Perfluoropentanoic acid, PFPeA, C5	2.07
Perfluorobutyric acid, PFBA, C4	3.03
Perfluoropropionic acid, PFPrA, C3	2.10
Trifluoroacetic acid, TFA, C2	2.13
Total PFCAs	20.99
